# Occupational Self-Efficacy as a Mediator in the Reciprocal Relationship between Job Demands and Mental Health Complaints: A Three-Wave Investigation

**DOI:** 10.3390/ijerph182111532

**Published:** 2021-11-02

**Authors:** Jasmina Tomas

**Affiliations:** Department of Psychology, Faculty of Humanities and Social Sciences, University of Zagreb, 10000 Zagreb, Croatia; jasmina.tomas@ffzg.hr

**Keywords:** job demands, mental health, mediation, occupational self-efficacy, job demands-resources theory, social cognitive theory, longitudinal study

## Abstract

The most recent version of the job demands-resources (JD–R) theory proposes that demanding working conditions and employee strain form a self-perpetuating loss cycle. By acknowledging that such cycles are detrimental for both employees and organizations, the present study aimed to contribute to enhancing the current scarce understanding regarding their explanatory mechanisms. For this purpose, it applied social cognitive theory to propose that occupational self-efficacy mediates the effects of two role stressors (i.e., role ambiguity and role conflict) on employee mental health complaints and vice versa. The hypothesized reciprocal mediation effects were tested using a three-wave full panel research design and a dataset of 917 (N_T1_ = 513, N_T1+T2_ = 122, N_T1+T3_ = 70, N_T1+T2+T3_ = 212) Croatian employees working in heterogeneous private sector industries. The results demonstrated that role conflict, but not role ambiguity, undermined employees’ beliefs in their capabilities to successfully master their jobs which, in turn, led them to experience more mental health complaints over time. Contrary to expectations, poor mental health did not lead to diminished efficacy beliefs nor, in turn, more job demands over time. Overall, the results of this study demonstrated an additional mechanism in the job demands-strain relationship and, at the same time, shed new light on the role of personal resources within the JD–R theory. Accounting for the malleable nature of employee efficacy beliefs, the study proposes several ways in which organizations can enhance occupational self-efficacy and thereby curb the causal chain linking job demands and employee strain reactions.

## 1. Introduction

Today we know very well that certain job characteristics, such as work overload and role ambiguity, can cause stress and harm employees’ health. When framed within one of the leading job stress theories—the job demands–resources (JD–R) theory—these job characteristics represent job demands or “those physical, psychological, social or organizational aspects of the job that require sustained physical and/or psychological effort and are therefore associated with certain physiological and/or psychological costs” [1] (p. 274). The most recent version of the JD–R theory also accounts for growing evidence of reversed effects in which employees who experience high levels of stress and health issues may perceive and generate more job demands over time [1,2]. In this way, employees may find themselves caught in loss cycles, in which job demands lead to strain, which in turn again leads to job demands [3]. Loss cycles are not only detrimental for individual employees but can also undermine the effectiveness of entire organizations. For example, chronically exhausted employees tend to perform poorly, spark conflicts with their colleagues and create confusion about work-related assignments [4], which can spill over onto the performance of entire organizations.

Although extant studies provide compelling empirical support for the existence and harmfulness of loss cycles [3], much less is known about (i) why job demands cause stress and impair employees’ health and conversely, (ii) why strain leads to higher levels of job demands? The lack of knowledge about the psychological processes underlying the reciprocal effects between job demands and strain has led the authors of the JD–R theory to label them as one of the “unsettled issues that need to be addressed” [1] (p. 277), as well as to call for research into the mediators that explain how vicious cycles can be broken [3]. Consistent with the central role mediators play in the development of psychological theories [5], knowing not just whether, but also why job demands and strain transmit the reciprocal effects between each other might further support and refine the JD–R theory because these theoretical explanations can provide a deeper and more nuanced understanding of the theory’s two main causal pathways [1]. Furthermore, such an understanding might guide practitioners to design more effective interventions [6] that will target the most susceptible parts of loss cycles.

Therefore, the aim of this study is to explore the mediating mechanism underlying the reciprocal effects between job demands and strain. To do so, the study comports with two suggestions outlined in the JD–R literature [1,3,7]. Firstly, it builds on the complementary theoretical framework—the social cognitive theory [8]—to advance occupational self-efficacy as a potential explanatory mechanism underlying loss cycles. Occupational self-efficacy concerns employees’ confidence in their ability to successfully perform a job and master various job-related challenges [9]. In line with the social cognitive theory, efficacy beliefs may be shaped by both the external work environment, such as job demands, and internal psychological states, such as employee strain [8]. In addition, efficacy beliefs may also influence (objective and perceived) external working conditions and employees’ psychological states [10]. Building on the equivalent theoretical propositions, the existing studies have demonstrated that employees’ self-efficacy plays a mediating role in gain cycles, i.e., cycles formed by job resources and motivational outcomes [11,12]. Job resources represent a second major category of job characteristics within the JD–R theory that are functional in achieving work goals, stimulating personal growth, learning, and development (e.g., job autonomy and supervisor support; [1]. For example, Llorens et al. [12] found that job resources increase employees’ efficacy beliefs which in turn enhance their work engagement, and vice versa, work engagement enhances self-efficacy, which in turn increases job resources. By integrating propositions derived from social cognitive theory and findings supporting the mediation role of efficacy beliefs in gain cycles, this study posits that: (i) job demands have the opposite, debilitating effects on occupational self-efficacy resulting in increased strain, and (ii) that strain undermines efficacy beliefs leading to higher levels of (perceived) job demands.

Secondly, the study uses a three-wave full panel design needed to test inherently causal mediation effects more accurately. The nascent studies examining intermediate mechanisms that link job demands and employee strain have thus far predominantly used cross-sectional research designs [13,14,15]), which cannot demonstrate that a predictor variable over time causes changes in the mediator and that a mediator further causes changes in an outcome variable [16]. Such methodological preconditions to a higher degree match the full panel research design with a minimum of three measurement occasions [16]. As another advantage, a three-wave full panel design allows examination of reciprocal mediation effects, which, compared to unidirectional effects, more accurately reflect propositions of social cognitive theory.

In all, this study has the potential to make the following contributions. First, it addresses one of the “unresolved issues” [1] (p. 277) in the JD–R theory, which concerns the underlying mechanisms of its causal pathways. In doing so, it contributes to the few studies examining the mediators underlying the effects of job demands on employee strain (cf. [1]), and to the even fewer studies examining the mediators underlying the effect of employee strain on job demands (cf. [3]). Second, by building on the social cognitive theory to frame occupational self-efficacy as a mediator, the study has the potential to shed new light on the role of personal resources in the job demands-strain relationship. Personal resources refer to individual characteristics linked to resiliency and perceived control over one’s environment [11]: accordingly, self-efficacy represents a prototypical example [1]. At present, the JD–R theory posits that personal resources act as buffers against the undesirable effects of job demands [3]. However, empirical support for this proposition is limited [1,17]. Therefore, this study proposes an alternative perspective according to which personal resources have the potential to transmit the undesirable influence of job demands onto employee strain and vice versa. Although the mediation role of personal resources in the job demands-strain relationship has previously been suggested by Taris and Schaufeli [18], its empirical examination has thus far been limited to only a few studies (e.g., [19]). Third, the study utilizes a three-wave full panel design which substantially improves the inferential power compared to predominantly used cross-sectional tests of mediation [16]. Fourth, this study aims to assist practitioners in developing effective organizational interventions. Although primary interventions that directly target job demands should be used as a most effective tool, many job demands often cannot be entirely eliminated from the work environment (e.g., emotional demands in the health care system). Therefore, interventions based on strengthening occupational self-efficacy may represent a promising alternative or supplemental route to forestall loss cycles between job demands and strain and promote individual and organizational well-being.

### 1.1. Job Demands and Strain in the Context of the Present Study

To conceptualize job demands, the study uses two role stressors—role ambiguity and role conflict—which are prototypical examples of job demands within the JD–R theory [1]. Numerous studies have shown that both role stressors have detrimental effects on employee well-being (for meta-analytical results, see [20]). Given their harmfulness and ubiquity in different occupations, focusing on these two job demands might advance practical interventions across a wide range of diverse work settings.

**Figure 1:**
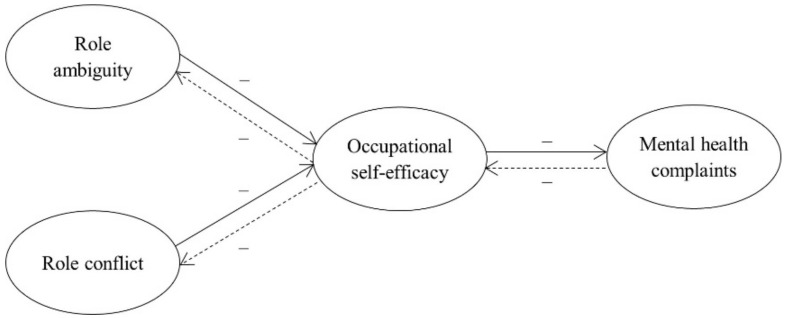
Hypothesized mediation model.

On the other hand, employee strain has been conceptualized as poor mental health, i.e., mental health complaints. According to the World Health Organization, mental health represents “a state of well-being in which an individual realizes his or her own abilities, can cope with the normal stresses of life, can work productively and is able to make a contribution to his or her community” [21]. The decision to focus on poor mental health as an indicator of employee strain was guided by its overarching negative impact on individuals and entire societies, particularly during the ongoing coronavirus pandemic. For example, research shows that poor mental health predicts the development of mental illnesses and suicides (e.g., [22]). At the organizational level, poor mental health has been associated with productivity loss expressed in billions of dollars (e.g., [23]). Given that the coronavirus pandemic has increased uncertainty, fear, and anxiety worldwide [24], preserving employees’ mental health is a particularly topical issue. The hypothesized research model is shown in Figure 1.

### 1.2. Reciprocal Relationships between Job Demands and Mental Health Complaints: Social Cognitive Theory as an Explanatory Framework

At present, the JD–R theory posits that job demands lead to poor health through a health-impairment process [1], according to which the demanding aspects of one’s job cost effort and consume energy resources [25]. Over time, exposure to job demands may lead to chronic exhaustion and, if prolonged, impaired mental and physical health [3]. This proposition has been supported by several studies (e.g., [26,27]). However, scholars have debated whether the energy-based explanation is comprehensive enough to account for the complex and overarching detrimental effects of job demands on employee health [1,7,18]. Consistent with this debate, the examples of alternative mediators tested in extant studies include satisfaction of the basic psychological needs [13], obsessive and harmonious passion [14], deep and surface acting [15], and recovery experiences [28]. On the other hand, currently proposed mechanisms of the effects of strain on job demands include maladaptive self-regulation strategies, such as self-undermining and coping inflexibility [29]. The present study aims to extend both lists in line with the following.

#### 1.2.1. The Mediated Effect of Role Ambiguity and Role Conflict on Mental Health Complaints

Building on social cognitive theory, this paper proposes that role ambiguity and role conflict have the potential to undermine employees’ occupational self-efficacy by conveying two sources of information regarding one’s work-related capabilities [30]. In the order of the expected strength of their influence, these are experiences of performance failures (pertaining to enactive mastery experiences) and evoked physiological and affective states, such as fatigue and anxiety (which partially overlap with the energy process proposed by the JD–R theory). However, this is not intended to imply that other job demands cannot provide additional or different sources of information for evaluating one’s job-related capabilities (e.g., through performance feedback) [8].

Role ambiguity reflects perceived uncertainty about various aspects of one’s job [31], such as ambiguous work responsibilities, goals, and scope of authority. Lack of clarity about these fundamental aspects of one’s work role may predispose an employee to experience repeated performance failures over time. Because people strongly rely on previous performance successes and failures when assessing their capabilities [8], failures stemming from prolonged exposure to ambiguous work roles are hypothesized to diminish occupational self-efficacy. Secondly, ambiguous circumstances may compel employees to invest additional effort in meeting their goals. Over time, these additional efforts may come with extra costs, leaving employees weary, frustrated, and anxious; a suggestion that aligns with the previous characterization of role ambiguity as “an inherently noxious state” [31] (p. 191). All of these are examples of physiological and affective states that employees tend to use to assess their personal efficacy, or in this case, inefficacy [8].

Complementary theoretical explanations may also be applied to predict how role conflict influences occupational self-efficacy. Role conflict occurs when employees need to deal with two or more incompatible demands associated with their work roles [32]. This paper focuses on one particular type of role conflict—a person-role conflict—which reflects the extent to which role demands are incompatible with an employee’s role expectations, internal standards, and values [32,33]. Given that characteristics such as one’s values are fairly stable and fundamental, their incongruity with role demands may lead to particularly severe health-related consequences, such as depression and coronary disease [34]. Comparable to role ambiguity, person-role conflict can potentially hamper employees’ performance successes and induce unpleasant physiological and affective states. For example, imagine a salesperson who is asked to sell a substantial amount of poor-quality product and is aware that this product is not something customers should spend their money on. Not only might this employee feel less willing and able to achieve this goal, but continuously coping with such a job demand may lead to feelings of fatigue, frustration, and anger [35], all of which are processes that tend to thwart sense of efficacy. Consistent with theoretical propositions delineating the harmful effects of both job demands, Rigotti et al. [36] found that overload from task demands related to decreased career self-efficacy over one year.

Moving forward in the hypothesized causal chain, this study suggests that reduced occupational self-efficacy may impair employees’ mental health. Bandura [8,10] provides several reasons why efficacy beliefs are a powerful determinant of people’s well-being. Applying his arguments to the work context, the perception of not being capable of successfully fulfilling job-related tasks is stressful and may deteriorate one’s mental health as employees may continuously feel overloaded by their task duties. On a related note, people with low efficacy beliefs tend to be less inventive in finding ways to exert control in their environments [10]. Because most people are motivated to feel in control, a perceived lack of it is unpleasant in and of itself and may have a harmful effect on the employees’ well-being [37]. Finally, as with other personal resources, occupational self-efficacy is linked to resiliency [38]. Employees with low efficacy tend to withdraw in the face of day-to-day difficulties and setbacks or, as Bandura [10] frames it, “slacken their efforts, give up prematurely, or settle for poorer solutions” (p. 180). Over time, these coping strategies may indeed place employees in less favorable positions and situations detrimental to their mental health. Empirical studies demonstrating the beneficial effects of efficacy beliefs on employee well-being are abundant. For example, Unsworth and Mason [39] have shown that self-efficacy-enhancing intervention reduces employee stain.

In summary, the pattern of assumptions derived from social cognitive theory provides a basis for arguing that diminished efficacy beliefs have the potential to mediate the detrimental effects of job demands on employee strain. Consistent with this proposition, Huang et al. [19] found that organization-based self-esteem and optimism, but not the general self-efficacy, mediated the detrimental effect of workload on employee burnout. This finding is not surprising because previous literature has treated self-esteem and optimism, as measured in Huang et al.’s study, as malleable characteristics open to development [10]. In comparison, the general self-efficacy, which concerns one’s beliefs about being capable of succeeding in different tasks across situations [40], has been conceptualized as a more stable trait-like characteristic less influenced by employees’ working conditions. Using the same sample and three measurement waves, Wang et al. [41] found that personal resources mediated the effect of job resources, but not the effect of job demands on burnout. However, it is worth noting that job demands and personal resources exerted a significant cross-lagged effect on personal resources and burnout, respectively, although not in a causal order that would confirm the mediation effect. To the best of the author’s knowledge, the study by Wang et al. [41] is the only study that examined the mediation role of personal resources in a job demands-strain relationship using a research design comparable to that used in the current study. However, these authors did not test the reversed mediation path, according to which employee strain leads to more job demands through diminished personal resources. As discussed in the following paragraphs, the social cognitive theory provides arguments for why this path should also be considered. In keeping with the presented theoretical and empirical arguments, the first hypothesis in this study is as follows:

**Hypothesis** **1** **(H1).** *Occupational self-efficacy mediates the relationship between role ambiguity (H1a) and role conflict (H1b) and subsequent mental health complaints such that higher levels of both role stressors relate to a decrease in occupational self-efficacy, and lower levels of occupational self-efficacy in turn relate to an increase in mental health complaints over time.*

#### 1.2.2. The Mediated Effect of Mental Health Complaints on Role Ambiguity and Role Conflict

As described earlier, people partially rely on their emotional states and mood when judging their own capabilities. Therefore, not only can a low sense of efficacy result in poor mental health, but mental health complaints can also diminish efficacy beliefs [8]. People with poor mental health experience negative moods, tension, anxiety, and feel dysfunctional in relation to others, all states that can easily be interpreted as signs of personal inefficacy [10]. In addition, these unpleasant states can evoke memories and thoughts about past failures, thus indirectly reducing self-efficacy [8]. In line with the assumptions that personal resources can function as both the antecedents and outcomes of mental health, Reis et al. [42] found that psychological capital predicted higher levels of mental health and that mental health predicted higher levels of psychological capital over time.

Applying social cognitive theory, it might also be suggested that occupational self-efficacy may further influence perceived levels of role ambiguity and role conflict. There are two plausible explanations for this effect. First, efficacy beliefs influence how people perceive their environments [8]. As such, employees who are more convinced of their abilities to master their jobs successfully may be more prone to interpret their work environments in a more favorable way or, in this particular case, perceive less ambiguity and conflict within their work role. Second, employees might play a more active role in relation to their environment by changing its objective features, a process that their self-efficacy may strongly influence. Those who are more confident in their job-related abilities might take a more proactive stance towards unfavorable working conditions and attempt to reduce job demands on their own. For example, highly efficacious employees might be more inclined to proactively ask for additional information that will help them better understand their duties or try to negotiate the content of their tasks so that it better fits with their values. These and similar proactive changes made by employees to reduce their job demands (and increase job resources) are known in the literature as job crafting [43]. In this regard, self-efficacy may not only determine in which behaviors employees will engage but also the amount of invested effort and perseverance [10]. This also implies that employees who think poorly about their capabilities are more likely to simply accept their unfavorable status quo. Alternatively, in a more extreme scenario, they might engage in self-undermining behaviors, i.e., self-destructive behaviors through which employees generate obstacles that undermine performance (e.g., by spurring confusion when communicating with others [44]). Over time, these behaviors may lead to higher job demands [4], including more ambiguous and incongruent work roles. The longitudinal beneficial effect of self-efficacy on the amount of job demands perceived and created by employees has been empirically demonstrated in previous studies (e.g., [45]).

In summary, this paper examines the assumption that employees with poorer mental health perceive higher levels of role ambiguity and role conflict because this unfavorable mental state undermines their belief that they are capable of successfully mastering their jobs. To the best of the author’s knowledge, this is the first study to test whether occupational self-efficacy mediates the effect of employee strain on job demands. However, as previously stated, existing studies have shown that positive affective states, such as work engagement, can lead to more job resources through increased efficacy beliefs [12]. Accordingly, the second hypothesis is as follows:

**Hypothesis** **2** **(H2).** *Occupational self-efficacy mediates the relationship between mental health complaints and subsequent role ambiguity (H2a) and role conflict (H2b) such that higher levels of mental health complaints relate to a decrease in occupational self-efficacy, and lower levels of occupational self-efficacy in turn relate to an increase in both role stressors over time.*

## 2. Materials and Methods

### 2.1. Procedure and Participants

Data were collected as part of a larger research project focusing on the relationships between the psychosocial work environment, employee personal resources and well-being. Participants were recruited from private sector organizations stationed across Croatia. The present study employed the sample recruited in the five largest organizations included in the project (i.e., two information technology (IT) companies, one insurance company, one medical equipment company, and one automobile company). Although diverse in terms of the industry, all companies operate in competitive and dynamic economic environments, which can be conducive for the development of occupational stress. A longitudinal study design comprised three measurement waves spaced approximately 6 months apart. The first wave took place from May to July 2016 (Time 1; T1), the second wave took place from November 2016 to January 2017 (Time 2; T2), and the third wave took place from May to July 2017 (Time 3; T3). The rationale for the choice of a 6-month time lag is provided in Supplementary Note S1 [46].

Data collection was organized in collaboration with HR managers. In exchange for their participation, each organization received a report with an analysis of psychosocial job characteristics. Data were collected via an online questionnaire. The introduction to the survey informed participants about the purpose of the study, the voluntary nature of their participation and the anonymity of the data. Employees were not compensated for their participation. However, several other literature-based techniques [47], reported in Supplementary Note S2, were used to increase response rates.

The invitation to participate in each survey was emailed to each employee, regardless of their participation in previous wave(s) [48]. A total of 1631 employees were invited to participate at T1, 1897 at T2, and 2061 at T3. Of those invited, 1220 employees began completing the survey at T1 (response rate of 75%), 1513 at T2 (response rate of 80%), and 1314 at T3 (response rate of 64%). Based on the data screening procedure, participants who did not meet the following criteria across three waves were excluded from the analyses: participants who filled out the survey more than once (determined by a combination of their identifier and background characteristics), participants who provided low-quality data (e.g., exhibited a pattern of a long string of “middle” responses [49]), and participants who reported an intra-organizational job transition between the three waves (as these changes may affect the examined cross-lagged relationships, [50]). Moreover, the analyses were based on data from participants who provided complete information about their T1 covariates. As a result, the effective longitudinal sample included 917 participants: 513 (55.9%) who participated at T1 only, 122 (13.3%) who participated at T1 and T2, 70 (7.6%) who participated at T1 and T3, and 212 (23.1%) who participated in all three measurement waves.

At T1, the average age of participants was 38.06 years (SD = 9.25). Approximately half of the sample were female (50.2%), and around two-thirds were highly educated (i.e., 63.2% held a Bachelor’s, Master’s, or postgraduate degree, whereas 36.8% had completed upper secondary or pre-university education). The vast majority of participants had a permanent contract (91.5%). Among the remaining 8.5%, 8.3% had a temporary contract, and 0.2% were employed over a specific type of temporary contract that enables occupational training without entering into an employment relationship and is intended to increase the employability of young employees who have just entered the labor market. The average organizational tenure was 81.56 months (SD = 70.10). Most of the participants (70.6%) did not hold a managerial position (and the positions of the remaining 29.4% of employees varied from lower to high managerial positions). Finally, the majority worked full-time (99.7%). An overview of the sample’s characteristics is presented in Supplementary Table S1.

To test whether there was any systematic drop out of participants, a binary logistic regression analysis was conducted. Specifically, participation at all three waves versus drop out at any wave was predicted by demographic and work-related variables (i.e., gender, age, education, contract type, and managerial position (Step 1)), and the focal study variables (i.e., role ambiguity, role conflict, occupational self-efficacy, and mental health complaints at T1 (Step 2)). Due to its high correlation with age (r = 0.56, *p* < 0.001), organizational tenure was not included simultaneously with age in this or any other analysis. Results demonstrated that Step 1 significantly contributed to predicting respondents’ dropout (χ^2^(5) = 22.67, *p* < 0.001): dropout was higher among male (OR = 0.57, *p* < 0.01) and less educated (OR = 1.56, *p* < 0.05) respondents. Importantly, Step 2 of the analysis was not statistically significant (χ^2^(4) = 8.03, *p* > 0.05), demonstrating that drop out at any wave was not dependent on perceived levels of the two role stressors, occupational self-efficacy and mental health complaints. In order to abate the likelihood of systematic drop out effects that could lead to biased parameters, missing data among all 917 respondents were treated using the full information maximum likelihood (FIML) estimation [51] (note that FIML was used to estimate the missing data both within and across measurement occasions). This procedure enabled the inclusion of participants who participated in one, two, or three measurement waves. The other advantage of FIML is that it produces more accurate standard errors of parameter estimates due to greater statistical power and thereby enables a more accurate test of the study hypotheses, particularly in comparison to listwise deletion [48].

### 2.2. Measures

Following the logic of the full panel design, each variable was measured at each of the three waves. All measures were subjected to a translation and back-translation procedure.

#### 2.2.1. Job Demands

Role ambiguity and role conflict were measured using the items that measure two corresponding subdimensions in the Psychological Climate Questionnaire originally developed by James and colleagues (e.g., [52]) and adapted by Tomas et al. [53]. Each job demand was measured with five items. Sample items for role ambiguity are: “It is often not clear who has the authority to make decisions regarding my job.” and “My work assignments are clearly defined.” (reversely coded). Sample items for role conflict are: “I have to do things that should be done differently.” and “A lot of what I do contradicts my personal attitudes.” Respondents were asked to indicate their responses on a 5-point Likert scale ranging from 1 (totally disagree) to 5 (totally agree). The Cronbach’s alpha coefficients were 0.84 (T1), 0.84 (T2), and 0.85 (T3) for role ambiguity and 0.87 (T1), 0.89 (T2), and 0.87 (T3) for role conflict.

#### 2.2.2. Occupational Self-Efficacy

Occupational self-efficacy was measured with the short six-item version of the occupational self-efficacy scale developed by Schyns and von Collani [9] and adapted by Rigotti et al. [54]. All items in this scale are tailored to the work context (e.g., “I meet the goals that I set for myself in my job”). Respondents rated each item on a scale ranging from 1 (not at all true) to 6 (completely true). The Cronbach’s alpha coefficients were comparable to the ones obtained by Rigotti et al. [54]: coefficients obtained in this study were 0.86 (T1), 0.84 (T2), and 0.89 (T3), and coefficients obtained by these authors ranged from 0.85 to 0.90.

#### 2.2.3. Mental Health Complaints

To measure mental health complaints in this study, four items of the Mental Health Inventory [55] were used. A sample item is “How much of the time, during the last month, have you been a very nervous person?” Responses were provided on a scale ranging from 0 (never) to 6 (always). The Cronbach’s alpha coefficients were 0.83 (T1), 0.85 (T2), and 0.84 (T3). As such, they were comparable to the ones obtained in previous studies. For example, in a recent study conducted among Romanian workers, Tisu et al. [56] reported a Cronbach alpha coefficient of 0.84.

#### 2.2.4. Control Variables

Previous studies showed that the variables of primary interest in this study could vary by gender [57], age [58], education [59], and managerial position [60]. Therefore, these sociodemographic variables, as well as organizational membership, were considered as potential confounding variables that could lead to spurious results. To avoid statistical overcontrol that can attenuate the effects of interest [16], only those variables with non-trivial relationships with core study variables were included in the analyses testing study hypotheses. Accounting for the correlations presented in Table 1, each of the study constructs was regressed on organizational membership variable; role ambiguity was additionally regressed on age and education, and occupational self-efficacy was additionally regressed on managerial position.

### 2.3. Analytical Procedure

The first step of data analyses involved testing the hypothesized measurement model and its invariance across time, whereas the second step entailed testing the hypothesized indirect effects.

The overall fit of the hypothesized measurement model was evaluated using confirmatory factor analysis (CFA). Items intended to measure role ambiguity, role conflict, occupational self-efficacy and mental health complaints were allowed to load onto their respective factor at each measurement occasion. The resulting 12 factors (i.e., four factors over three waves) were allowed to correlate with each other. Finally, to account for the temporal stability of the indicator-specific variance, correlations between item residuals were specified for each pair of equivalent items across time [61]. The hypothesized four-factor model was then tested against two alternative models (each specified at three waves simultaneously): a three-factor model in which indicators intended to measure role ambiguity and role conflict loaded on one factor and a one-factor model.

Prior to testing the study hypotheses, it was also necessary to demonstrate the longitudinal measurement invariance of the best-fitting measurement model. For this purpose, a series of three nested models, each imposing an increasing degree of invariance on model parameters across time, were compared: the configural invariance model (with factor structure constrained equal across time), the metric invariance model (with factor loadings additionally constrained equal across time), and the scalar invariance model (with item intercepts additionally constrained equal across time). Measurement invariance is demonstrated when the more constrained model (e.g., scalar invariance model) fits the data equally well as the less constrained model (e.g., metric invariance model). Without demonstrating at least partial longitudinal invariance of factor loadings and intercepts, it is impossible to know whether any changes observed in constructs are real changes or whether the meaning of the items changed across time [16]. In the latter case, the interpretability of the cross-lagged coefficients might be impeded.

The second step of the analyses involved testing the hypothesized indirect effects from job demands to mental health complaints through occupational self-efficacy (H1a and H1b, respectively) and, vice versa, from mental health complaints to job demands through occupational self-efficacy (H2a and H2b, respectively). The starting point for these analyses was the measurement model with factor structure, factor loadings, and intercepts constrained equal across time to the degree allowed by the longitudinal measurement invariance test. To test the hypothesized cross-lagged paths, the nondirectional cross-time relationships between the factors needed to be converted into directed relationships [16]. This was carried out in line with the logic of the four nested structural models: (i) the stability model (including only autoregressive paths); (ii) the normal causation model (including cross-lagged paths from role ambiguity and role conflict at T1 and T2 to occupational self-efficacy at T2 and T3, respectively; occupational self-efficacy at T1 and T2 to mental health complaints at T2 and T3, respectively; and role ambiguity and role conflict at T1 to mental health complaints at T3); (iii) the reversed causation model (including cross-lagged paths from mental health complaints at T1 and T2 to occupational self-efficacy at T2 and T3, respectively; occupational self-efficacy at T1 and T2 to role ambiguity and role conflict at T2 and T3, respectively; and mental health complaints at T1 to role ambiguity and role conflict at T3); and (iv) and the reciprocal causation model (i.e., the hypothesized model; including all cross-lagged paths from the normal causation model and the reversed causation model). Each structural model additionally included: control variables; correlations between error variances of the corresponding items across time; correlations between all exogenous variables at T1; and correlations between all disturbance factors associated with endogenous variables at T2 and T3 [16].

The final analysis in this step included a test of time invariance of the best fitting structural model. To do so, the paths from T1 to T2 were fixed to be equal to the equivalent paths from T2 to T3 in the following order: first, the autoregressive paths; then the path(s) from the predictor(s) to the mediator; and finally, the path(s) from the mediator to the outcome(s). Conclusions regarding the study hypotheses were based on the model allowing for the highest level of time invariance without compromising the model fit. The statistical significance of the indirect effects was estimated from the 95% confidence intervals (CIs) provided by bootstrap analysis (10,000 resamples).

All measurement and structural models were tested in the Mplus 8.5 statistical software [62] using maximum likelihood estimation. In evaluating the goodness-of-model fit, well-established cut-off criteria were applied (i.e., CFI and TLI > 0.90; RMSEA and SRMR < 0.08, [63,64]). Nested models were statistically compared with the conventionally used chi-square difference test (Δχ^2^). However, this test has been criticized as being too stringent to serve as an indicator of measurement invariance due to its sensitivity to sample size [65]. Therefore, measurement invariance was also evaluated based on the difference in CFI (ΔCFI). According to this indicator, non-invariance is established when the change in CFI between two nested models exceeds a value of 0.01 [65].

## 3. Results

Mean values, standard deviations, Cronbach’s alpha coefficients and correlations for all study variables are presented in Table 1.

### 3.1. Testing the Measurement Model and Longitudinal Measurement Invariance

The hypothesized measurement model consisting of four factors at three waves demonstrated a satisfactory fit to the data (χ^2^ = 2811.052, df = 1584, *p* < 0.001, CFI = 0.928, TLI = 0.920, RMSEA = 0.029, SRMR = 0.054). All items were strongly related to the respective latent factor (with standardized factor loadings ranging from λ = 0.57 to 0.87). Furthermore, this model fitted the data significantly better than an alternative three-factor model in which items intended to measure role ambiguity and role conflict loaded onto one factor at each wave (Δχ^2^ = 1188.115, Δdf = 30, *p* < 0.001, CFI = 0.860, TLI = 0.847, RMSEA = 0.040, SRMR = 0.065), and a one-factor model in which all items loaded onto one factor at each wave (Δχ^2^ = 5409.300, Δdf = 63, *p* < 0.001, CFI = 0.615, TLI = 0.586, RMSEA = 0.066, SRMR = 0.118).

The satisfactory model fit of the hypothesized four-factor model (i.e., a configural invariance model) supported the notion of equal factor structures across three waves. Further fixing of model parameters equal across time did not compromise a model fit: neither fixing of factor loadings in a metric invariance model (Δχ^2^ = 39.581, Δdf = 32, *p* > 0.05, ΔCFI = 0), nor of item intercepts in a scalar invariance model (Δχ^2^ = 44.762, Δdf = 32, *p* > 0.05, ΔCFI = 0.001). Accordingly, the assumptions of full metric and scalar invariance were supported, enabling a valid test of the study hypotheses in the following step. A more detailed overview of model fit indices is presented in Table 2.

### 3.2. Testing the Hypothesized Indirect Effects

Statistical comparison of structural models demonstrated that the normal causation model outperformed the three remaining competing models. First, only the normal and reciprocal causation models fitted the data significantly better than the stability model (Δχ^2^ = 23.612, Δdf = 8, *p* < 0.01 and Δχ^2^ = 32.012, Δdf = 16, *p* < 0.01, respectively). Second, the reciprocal causation model did not fit the data better than the normal causation model (Δχ^2^ = 8.400, Δdf = 8, *p* > 0.05). Hence, because the more parsimonious model—the normal causation model—was found to be the baseline model for further testing of indirect effects, hypotheses H2a and H2b were immediately refuted. An overview of fit indices of each structural model is presented in Table 2.

To further examine whether the structural paths of the normal causation model were invariant across time, the autoregressive paths were fixed as equal between the three waves. This constrained model did not significantly deteriorate the model fit (Δχ^2^ = 2.839, Δdf = 4, *p* > 0.05). Therefore, analysis proceeded by fixing the paths from role ambiguity and role conflict to occupational self-efficacy as equal across time (i.e., “a” paths in the mediation model). The results demonstrated that the paths from these two predictor variables to the mediator were stable across time (Δχ^2^ = 1.258, Δdf = 2, *p* > 0.05). The same conclusion was drawn regarding the path from occupational self-efficacy to mental health complaints (i.e., “b” path in the mediation model): fixing these paths as equal across time did not significantly compromise the model fit (Δχ^2^ = 0.007, Δdf = 1, *p* > 0.05). To summarize, the results demonstrated that all structural paths were stable across time (see Table 2). Therefore, the constrained normal causation model presented in Supplementary Figure S1 was used as a basis from which to test whether occupational self-efficacy mediated the effects from role ambiguity and role conflict to mental health complaints.

The individual cross-lagged coefficients revealed the following results. Role ambiguity did not relate significantly to subsequent occupational self-efficacy (B = 0.09, *p* = 0.08). In contrast and as expected, role conflict did have a negative cross-lagged effect on employees’ efficacy beliefs (B = −0.15, *p* < 0.001). Occupational self-efficacy, in turn, related negatively to mental health complaints over time (B = −0.14, *p* = 0.04), which was also in line with expectations. Consistent with this pattern of findings, occupational self-efficacy did not mediate the effect of role ambiguity on mental health complaints (B = −0.01, 95% CI [−0.039, 0.00]), thus refuting H1a. However, in support of H1b, the indirect effect of role conflict on mental health complaints through occupational self-efficacy was significant and in the expected direction (B = 0.02, 95% CI [0.003, 0.055]): employees who experienced more role conflict over time became less confident that they were capable to successfully perform their job, and these decreased efficacy beliefs subsequently led them to experience more mental health complaints. Therefore, H1 was partially supported.

## 4. Discussion

The present study seeks to explain the psychological process underlying the reciprocal relationships between job demands and employee strain. By applying social cognitive theory [8], occupational self-efficacy is advanced as an explanatory mechanism underlying both causal relationships. The results provided partial support for the proposed process according to which detrimental working conditions deteriorate mental health by thwarting employee efficacy beliefs. However, they did not comport with the hypotheses delineating reversed mediation paths according to which mental health complaints lead to higher levels of job demands through diminished occupational self-efficacy. The following paragraphs discuss the plausible explanations for these findings.

First, the results show that occupational self-efficacy mediates the effect of role conflict but not the effect of role ambiguity on employee mental health complaints. These disparate results reflect the finding according to which role ambiguity did not exert the hypothesized detrimental effect on employees’ efficacy beliefs, whereas role conflict did lead to decreased occupational self-efficacy over time. To explain the disparate findings that lent empirical support for the suggestion that the person-role conflict (but not role ambiguity) thwart employees’ mastery experiences and evoke unpleasant physiological and affective states [8], it might be necessary to take a more nuanced perspective on each job demand and how these demands were experienced by the employees who participated in this study. Once again, this can be achieved by integrating theoretical propositions derived from the JD–R theory [1] and social cognitive theory [8].

Specifically, the more recent literature on the JD–R theory acknowledges that not all job demands necessarily thwart employees’ mastery experiences, personal growth, and goal attainment. Rather, some job demands can achieve the opposite by promoting mastery, learning, and future gains [35]. In line with this conceptual distinction, growing empirical evidence substantiates the difference between hindrance and challenge job demands (e.g., [66]). Both types of job demands require effort and, as a result, consume employees’ energy resources [1]. However, hindrance job demands are perceived as constraints that unnecessarily interfere with employees’ progress towards goal achievement, whereas challenge demands are experienced as opportunities for learning, task achievement and personal growth [67]. Consistent with its prevailing status as a hindrance demand in the literature, the results indicate that person-role conflict was indeed perceived as an obstacle that interfered with employees’ task achievements, thereby hampering their mastery experiences [35]. These experiences have, in turn, provided information cues for employee inefficacy [30].

On the other hand, although it did not reach statistical significance, the positive sign of the effect of role ambiguity on occupational self-efficacy suggests that this job demand might have been experienced as a challenge rather than a hindrance demand for some employees in this study. This suggestion aligns with the JD–R theory and the transactional theory of stress, which both acknowledge that a single job demand may be experienced as a hindrance or a challenge demand depending on the context and the characteristics of the person who is appraising the demand [1,68]. The sample in this study was predominantly composed of highly educated employees performing highly skilled, non-manual jobs. These characteristics might have predisposed employees to perceive ambiguous goals, assignments, and authority boundaries as surmountable obstacles that could be mastered with additional effort. According to social cognitive theory, the most resilient sense of self-efficacy is established when individuals overcome setbacks and difficulties “through perseverant effort” and not when they experience “only easy successes” [10] (p. 185). Therefore, for highly skilled and educated employees, role ambiguity might have represented a stimulating challenge: by investing their energy into structuring ambiguous work roles requirements, employees were able to put their competence to good use and demonstrate their capabilities. For this reason, once role ambiguity was overcome, it might have promoted a sense of achievement, mastery, and a positive mood. The presented arguments are consistent with the study by Ventura et al. [69], who found that role ambiguity negatively relates to professional self-efficacy among the sample of secondary school teachers but not among a heterogeneous sample of ICT users.

Regarding the effect of occupational self-efficacy on mental health complaints, the results are consistent with the tenets of social cognitive theory and existing studies (e.g., [39]). Specifically, the study shows that employees with a more positive outlook about their abilities to master their jobs successfully reported fewer mental health complaints over time. A strong sense of occupational self-efficacy may thus have served as a protective factor that led employees to adopt a more proactive stance vis-à-vis everyday hassles at work and exercise control in their work environment, thereby reducing their stress levels and enhancing mental well-being.

In all, the results pertaining to the first two hypotheses complement the few previous studies that have included efficacy beliefs in the causal link together with demanding working conditions and employee well-being (e.g., [19,69]). Of note is that some of these studies posited that job demands explain the link between self-efficacy and well-being, and not the other way around [45,69]. Unfortunately, the research designs used to verify this assumption included a maximum of two measurement waves, which is suboptimal for a test of mediation. In contrast, the present study partially demonstrated that job demands (as elements of the external work environment) present a more distal antecedent of employee strain reactions, while employees’ efficacy beliefs (as internal personal resources) act as a more proximal antecedent of these reactions.

The second set of hypotheses suggested that employees with poorer mental health are more likely to evaluate their capabilities as inadequate for the successful accomplishment of job-related duties [8]. Lower levels of efficacy beliefs were, in turn, expected to prompt employees to perceive their work roles as more ambiguous and incongruent or even to behave in a way that would create such working conditions. However, the results did not lend support to these hypotheses. To better understand the plausible reasons for the nonsignificant indirect effects, it might be worthwhile to consider once again the assumptions of social cognitive theory in combination with the methodological aspects of this study.

Bandura [8] argued that it is not merely the intensity of emotional states and mood that is crucial for efficacy judgements, but rather how one interprets and integrates these experiences into their own efficacy beliefs system. As previously discussed, the characteristics of the person performing such cognitive processes determine the manner in which information conveyed by various sources of efficacy beliefs will be integrated into that person’s system of efficacy beliefs. As the sample in this study was mainly composed of highly skilled professionals, it is perhaps possible that higher levels of mental health complaints were not powerful enough to dimmish their efficacy beliefs. This argument might be substantiated by the observed negatively skewed distribution of occupational self-efficacy, which is indicative of a strong, more robust sense of efficacy beliefs. Moreover, a period longer than 6 months might have been necessary for poor mental health to instill the hypothesized detrimental process, such as that applied in previous studies [36,45]. Finally, the available research suggests that mastery experiences are more influential in shaping self-efficacy than internal states such as mood [70]. As such, it might be plausible that, when these factors were tested simultaneously in a reciprocal model, the mastery experiences conveyed by person-role conflict predominated over the effects of mental health complaints, thus rendering the latter nonsignificant in influencing occupational self-efficacy.

### 4.1. Theoretical and Practical Implications

Taken together, the results of this study have several implications for the JD–R theory. Firstly, the study indicates that employee efficacy beliefs can explain the link between job demands and strain reactions: demanding working conditions can instill self-doubt about one’s job-related capabilities, which in turn has long-term detrimental effects on mental well-being. As such, the study extends the list of previously examined mediators of the job demands-strain relationship by adding a plausible mechanism derived from social cognitive theory. However, the results also show that occupational self-efficacy cannot serve as a universal mechanism that accounts for the detrimental effects of each job demand (cf. [18]) or for the reversed effect starting from employee strain. In this regard, more precise predictions can potentially be achieved by accounting for employee work-related characteristics (e.g., skill level, education) as the potential moderators of the effects of job demands and strain on efficacy beliefs.

Furthermore, the study sheds new light on the role of personal resources within the JD–R theory. In addition to their well-established mediating role in the motivation process and partially supported moderating role in the job demands-strain relationship, the results demonstrate that malleable personal resources can also explain why certain job characteristics harm employee mental health.

Accordingly, this study also indicates that fostering efficacy beliefs is one way in which HR practitioners might increase the effectiveness of interventions aiming to forestall loss cycles and enhance employee mental health. This suggestion becomes even more relevant in situations in which eliminating job demands from the work environment is challenging, as is often the case with person-role conflict. To do so, practitioners might benefit from applying social cognitive theory, which provides well-developed principles for governing the development of efficacy beliefs in a work context.

For example, one of the most effective ways organizations can cultivate a sense of efficacy among employees is to create working conditions that enable them to achieve graduated successes and learn how to overcome obstacles and failures through perseverant effort [10]. In this way, the resulting increases in occupational self-efficacy should be resilient to future failures and setbacks. The remaining principles include enabling employees to observe competent models similar to themselves, providing informative and encouraging feedback, and instructing employees on methods for reducing unpleasant physical and emotional states [10].

### 4.2. Limitations and Suggestions for Future Research

The first limitation concerns the use of a 6-month time lag, which was optimal for demonstrating the hypothesized indirect effect of at least one job demand on mental health but potentially suboptimal for detecting the reversed indirect effect. To examine whether occupational self-efficacy or similar personal resources can indeed account for the effects of poor mental health on increases in (perceived) job demands, longer time lags may be needed. This suggestion is in line with the work of Rigotti et al. [36], who found that emotional strain exerts a negative effect on career-self-efficacy after a period of 1 year, and Vera et al. [45], who demonstrated the negative effect of occupational self-efficacy on job demands using an 8-month time lag. Future studies may also examine whether the length of what is considered an optimal time lag for any of the hypothesized effects depends on employees’ human capital (e.g., level of professional skills). For example, longer time lags may be needed for mental health complaints to instill the hypothesized loss cycles among better-equipped individuals less prone to losses in efficacy beliefs, while the opposite might be true for ill-equipped employees.

Second, the sample in this study was not representative of the Croatian workforce, thus limiting the generalizability of the results. The composition of the sample in terms of their professional profiles may also have induced bias into the study results by underestimating the obtained effects due to a restriction of range in the mediator variable. Consistent with this suggestion is the relatively small obtained effect size of the indirect effect. As such, future studies might benefit from employing more heterogeneous and more representative samples.

A final limitation refers to the conceptualization of job demands, personal resources and strain. The choice of two role stressors in this study was based on their well-established harmfulness and omnipresence in various occupations and work settings. However, it might be interesting to examine whether job demands from other domains (e.g., interpersonal conflicts, emotional demands, and job insecurity) can instigate the loss of personal resources, in turn leading to more strain. Similar reasoning applies to personal resources and employee strain. Examples of alternative conceptualizations include but are not limited to psychological capital [71], organization-based self-esteem [72], as well as physical health complaints.

## 5. Conclusions

Altogether, the results of this study indicate that strengthening employees’ efficacy beliefs may generate an additional benefit that has been mainly overlooked in previous research: it may curb the causal chain linking the job demands and the employee strain reactions. Preserving employee mental health in the workplace is not only a worthwhile pursuit on its own, but also enables organizations to save substantial financial resources that are otherwise lost due to absenteeism and decreased productivity [73]. This may be particularly true in light of the ongoing COVID-19 pandemic when employees worldwide experience additional demands and threats at their workplace, ranging from direct exposure to the virus to increased job insecurity and conflicts between work and family demands [74,75]. Therefore, now, and perhaps more than ever in modern history, investing in employee mental health should be one of the most highly prioritized organizational goals.

## Figures and Tables

**Table 1:** Descriptive statistics, correlations, and Cronbach’s alpha coefficients (in parentheses).

Variables	M	SD	1.	2.	3.	4.	5.	6.	7.	8.	9.	10.	11.	12.	13.	14.	15.	16.
1. Gender	-	-	-															
2. Age (in years)	38.06	9.25	0.01	-														
3. Education	-	-	0.15 ***	−0.26 ***	-													
4. Contract type	-	-	−0.07 *	−0.30 ***	0.09 **	-												
5. Tenure (in months)	81.56	70.10	−0.05	0.56 ***	−0.16 ***	−0.27 ***	-											
6. Managerial position	-	-	0.06	0.12 ***	0.13 ***	−0.11 **	0.26 ***	-										
7. Org1	-	-	−0.20 ***	0.31 ***	−0.30 ***	−0.01 *	0.16 ***	−0.09 **	-									
8. Org2	-	-	0.27 ***	−0.17 ***	0.40 ***	−0.12 ***	−0.07 *	−0.04	−0.13 ***	-								
9. Org3	-	-	−0.05	−0.30 ***	0.20 ***	0.43 ***	−0.26 ***	0.08 *	−0.25 ***	−0.24 ***	-							
10. Org4	-	-	0.11 **	0.08 *	−0.22 ***	−0.07 *	0.06	0.08 *	−0.23 ***	−0.23 ***	−0.10 **	-						
11. Role ambiguity (T1)	2.35	0.72	0.05	−0.08 *	0.18 ***	−0.01 *	−0.01	0.05	−0.11 **	0.27 ***	0.03	−0.18 ***	(0.84)					
12. Role ambiguity (T2)	2.34	0.69	−0.03	−0.19 ***	0.21 ***	0.05	−0.06	0.00	−0.15 **	0.28 ***	0.05	−0.21 **	0.71 ***	(0.84)				
13. Role ambiguity (T3)	2.33	0.68	0.12 *	−0.21 ***	0.11	0.01	−0.10	0.00	−0.23 ***	0.29 ***	0.08	−0.15 *	0.67 ***	0.69 ***	(0.85)			
14. Role conflict (T1)	2.45	0.78	0.00	0.07 *	−0.02	0.01	0.06	−0.05	0.16 ***	0.01	−0.07 *	−0.15 ***	0.58 ***	0.47 ***	0.42 ***	(0.87)		
15. Role conflict (T2)	2.40	0.79	−0.10	−0.09	0.02	0.10	0.01	−0.07	0.11	0.02	−0.04	−0.16 **	0.51 ***	0.62 ***	0.45 ***	0.72 ***	(0.89)	
16. Role conflict (T3)	2.36	0.71	0.03	−0.08	−0.06	0.07	0.02	−0.05	0.02	0.04	−0.04	−0.09	0.46 ***	0.45 ***	0.57 ***	0.63 ***	0.71 ***	(0.87)
17. OCCSE (T1)	5.05	0.57	0.06	0.02	0.00	0.00	0.07 *	0.09 **	−0.15 ***	−0.05	0.01	0.19 ***	−0.26 ***	−0.22 ***	−0.29 ***	−0.21 ***	−0.17 **	−0.25 ***
18. OCCSE (T2)	5.04	0.50	0.07	0.11 *	−0.02	−0.05	0.05	0.14 *	−0.08	−0.07	−0.05	0.29 ***	−0.20 ***	−0.30 ***	−0.30 ***	−0.23 ***	−0.24 **	−0.23 ***
19. OCCSE (T3)	5.04	0.59	0.03	0.01	0.02	0.00	−0.02	0.11	−0.13 *	−0.02	0.06	0.29 ***	−0.20 **	−0.31 ***	−0.22 ***	−0.23 ***	−0.33 ***	−0.31 ***
20. MHC (T1)	2.13	0.97	−0.05	0.01	−0.04	−0.02	0.08 *	0.00	0.13 ***	−0.03	−0.01	−0.08 *	0.34 ***	0.31 ***	0.25 ***	0.47 ***	0.34 ***	0.29 ***
21. MHC (T2)	2.06	0.97	−0.015 **	−0.11 *	−0.02	0.00	0.02	−0.07	0.02	0.03	0.04	−0.05	0.34 ***	0.46 ***	0.31 ***	0.39 ***	0.49 ***	0.36 ***
22. MHC (T3)	2.07	0.95	−0.03	−0.10	−0.10	0.00	−0.01	−0.03	0.08	0.02	0.03	−0.15 *	0.29 ***	0.32 ***	0.33 ***	0.32 ***	0.30 ***	0.26 ***
Variables	17.	18.	19.	20.	21.	22.												
17. OCCSE (T1)	(0.86)																	
18. OCCSE (T2)	0.68 ***	(0.84)																
19. OCCSE (T3)	0.64 ***	0.52 ***	(0.89)															
20. MHC (T1)	−0.36 ***	−0.37 ***	−0.26 ***	(0.83)														
21. MHC (T2)	−0.36 ***	−0.38 ***	−0.32 ***	0.63 ***	(0.85)													
22. MHC (T3)	−0.36 ***	−0.39 ***	−0.19 ***	0.62 ***	0.65 ***	(0.84)												

Note: *N* = 917 (missing data were treated using the FIML estimation). Gender (0 = female, 1 = male), education (0 = upper secondary or pre-university education, 1 = Bachelor’s, Master’s, or postgraduate degree), contract type (0 = permanent, 1 = temporary), managerial position (0 = no, 1 = yes), Org1–Org4 (dummy recoded organizational membership), OCCSE = occupational self-efficacy, MHC = mental health complaints. * *p* < 0.05; ** *p* < 0.01, and *** *p* < 0.001.

**Table 2:** Fit indices and results of the comparison of competing nested measurement and structural models.

Model No.	Model Description	χ^2^	*df*	CFI	TLI	RMSEA	SRMR	Δ Model	Δχ^2^	Δ*df*
M1	Configural	2811.052 ***	1584	0.928	0.920	0.029	0.054	–	–	–
M2	Metric	2850.633 ***	1616	0.928	0.921	0.029	0.057	M2–M1	39.581	32
M3	Scalar	2895.395 ***	1648	0.927	0.921	0.029	0.057	M3–M2	44.762	32
S1	Stability	3626.510 ***	2051	0.910	0.904	0.029	0.072	–	–	–
S2	Normal causation	3602.898 ***	2043	0.911	0.905	0.029	0.064	S2–S1	23.612 **	8
S3	Reversed causation	3616.806 ***	2043	0.910	0.904	0.029	0.065	S3–S1	9.704	8
S4	Reciprocal causation	3594.498 ***	2035	0.911	0.904	0.029	0.061	S4–S1	32.012 **	16
								S4–S2	8.400	8
S5	Autoregressive paths constrained equal	3605.737 ***	2047	0.911	0.905	0.029	0.064	S5–S2	2.839	4
S6	Paths from RA and RC → OCCSE fixed equal	3606.995 ***	2049	0.911	0.905	0.029	0.064	S6–S5	1.258	2
S7	Path from OCCSE → MHC fixed equal	3607.002 ***	2050	0.911	0.905	0.029	0.064	S7–S6	0.007	1

Note: *N* = 917 (missing data were treated using the FIML estimation). RA = role ambiguity, RC = role conflict, OCCSE = occupational self-efficacy, MHC = mental health complaints. CFI = Comparative Fit Index, TLI = Tucker-Lewis Index, RMSEA = root mean squared error of approximation, SRMR = standardized root mean squared residual. ** *p* < 0.01, *** *p* < 0.001.

## Data Availability

Corresponding analyses can be requested from the author. The data cannot be shared publicly due to the initial legal agreements with participating organizations. For all data-related requests, please contact the author of the manuscript.

## Supplementary Materials

The following are available online at https://www.mdpi.com/article/10.3390/ijerph182111532/s1, Note S1: The rationale for the choice of a 6-month time lag, Note S2: Description of techniques used to increase response rates, Table S1: Sample’s demographic and work-related characteristics, Figure S1: Unstandardized path coefficients and confidence intervals.

## References

[1] Bakker A.B., Demerouti E. (2017). Job demands–resources theory: Taking stock and looking forward. J. Occup. Health Psychol..

[2] De Lange A.H., Taris T.W., Kompier M.A.J., Houtman I.L.D., Bongers P.M. (2004). The relationships between work characteristics and mental health: Examining normal, reversed and reciprocal relationships in a 4-wave study. Work Stress.

[3] Bakker A.B., Demerouti E., Diener E., Oishi S., Tay L. (2018). Multiple levels in job demands-resources theory: Implications for employee well-being and performance. Handbook of Well-Being.

[4] Bakker A.B., Wang Y. (2020). Self-undermining behavior at work: Evidence of construct and predictive validity. Int. J. Stress Manag..

[5] Memon M.A., Cheah J., Ramayah T., Ting H., Chuah F. (2018). Mediation analysis issues and recommendations. J. Appl. Struct. Equ. Model..

[6] MacKinnon D.P., Luecken L.J. (2008). How and for whom? Mediation and moderation in health psychology. Health Psychol..

[7] Schaufeli W.B., Taris T.W., Bauer G., Hämmig O. (2014). A critical review of the Job Demands-Resources Model: Implications for improving work and health. Bridging Occupational, Organizational and Public Health.

[8] Bandura A. (1997). Self-Efficacy: The Exercise of Control.

[9] Schyns B., von Collani G. (2002). A new occupational self-efficacy scale and its relation to personality constructs and organizational variables. Eur. J. Work Organ. Psychol..

[10] Bandura A., Weingart L., Jehn K.A. (2009). Cultivate Self-Efficacy for Personal and Organizational Effectiveness. Handbook of Principles of Organizational Behavior.

[11] Xanthopoulou D., Bakker A.B., Demerouti E., Schaufeli W.B. (2007). The role of personal resources in the job demands-resources model. Int. J. Stress Manag..

[12] Llorens S., Schaufeli W., Bakker A., Salanova M. (2007). Does a positive gain spiral of resources, efficacy beliefs and engagement exist?. Comput. Hum. Behav..

[13] Van den Broeck A., Vansteenkiste M., De Witte H., Lens W. (2008). Explaining the relationships between job characteristics, burnout, and engagement: The role of basic psychological need satisfaction. Work Stress.

[14] Trépanier S.-G., Fernet C., Austin S., Forest J., Vallerand R.J. (2014). Linking job demands and resources to burnout and work engagement: Does passion underlie these differential relationships?. Motiv. Emot..

[15] Peng K.Z., Wong C.-S., Che H.-S. (2010). The missing link between emotional demands and exhaustion. J. Manag. Psychol..

[16] Little T.D. (2013). Longitudinal Structural Equation Modeling.

[17] Kim S., Wang J. (2018). The role of job demands–resources (JDR) between service workers’ emotional labor and burnout: New directions for labor policy at local government. Int. J. Environ. Res. Public Health.

[18] Taris T.W., Schaufeli W.B., Clarke S., Probst T.M., Guldenmund F., Passmore J. (2016). The job demands-resources model. The Wiley Blackwell Handbook of the Psychology of Occupational Safety and Workplace Health.

[19] Huang J., Wang Y., You X. (2016). The job demands-resources model and job burnout: The mediating role of personal resources. Curr. Psychol..

[20] Schmidt S., Roesler U., Kusserow T., Rau R. (2014). Uncertainty in the workplace: Examining role ambiguity and role conflict, and their link to depression—A meta-analysis. Eur. J. Work Organ. Psychol..

[21] World Health Organization Mental Health: Strengthening our Response. https://www.who.int/news-room/fact-sheets/detail/mental-health-strengthening-our-response.

[22] Keyes C.L., Dhingra S.S., Simoes E.J. (2010). Change in level of positive mental health as a predictor of future risk of mental illness. Am. J. Public Health.

[23] Lee Y.C., Chatterton M.L., Magnus A., Mohebbi M., Le L.K.D., Mihalopoulos C. (2017). Cost of high prevalence mental disorders: Findings from the 2007 Australian National Survey of Mental Health and Wellbeing. Aust. New Zealand J. Psychiatry.

[24] Jokic-Begic N., Lauri Korajlija A., Mikac U. (2020). Cyberchondria in the age of COVID-19. PLoS ONE.

[25] Bakker A.B., Demerouti E., Sanz-Vergel A.I. (2014). Burnout and work engagement: The JD–R approach. Annu. Rev. Organ. Psychol. Organ. Behav..

[26] Hakanen J.J., Schaufeli W.B., Ahola K. (2008). The Job Demands-Resources model: A three-year cross-lagged study of burnout, depression, commitment, and work engagement. Work Stress.

[27] Hakanen J.J., Bakker A.B., Schaufeli W.B. (2006). Burnout and work engagement among teachers. J. Sch. Psychol..

[28] Kinnunen U., Feldt T., Siltaloppi M., Sonnentag S. (2011). Job demands–resources model in the context of recovery: Testing recovery experiences as mediators. Eur. J. Work. Organ. Psychol..

[29] Bakker A.B., de Vries J.D. (2021). Job demands–resources theory and self-regulation: New explanations and remedies for job burnout. Anxiety Stress Coping.

[30] Gist M.E., Mitchell T.R. (1992). Self-efficacy: A theoretical analysis of its determinants and malleability. Acad. Manag. Rev..

[31] Breaugh J.A., Colihan J.P. (1994). Measuring facets of job ambiguity: Construct validity evidence. J. Appl. Psychol..

[32] Rizzo J.R., House R.J., Lirtzman S.I. (1970). Role conflict and ambiguity in complex organizations. Adm. Sci. Q..

[33] Kahn R.L., Wolfe D.M., Quinn R.P., Snoek J.D., Rosenthal R.A. (1964). Organizational Stress: Studies in Role Conflict and Ambiguity.

[34] Latack J.C. (1981). Person/role conflict: Holland’s model extended to role-stress research, stress management, and career development. Acad. Manag. Rev..

[35] Crawford E.R., LePine J.A., Rich B.L. (2010). Linking job demands and resources to employee engagement and burnout: A theoretical extension and meta-analytic test. J. Appl. Psychol..

[36] Rigotti T., Korek S., Otto K. (2020). Career-related self-efficacy, its antecedents and relationship to subjective career success in a cross-lagged panel study. Int. J. Hum. Resour. Manag..

[37] Bordia P., Hunt E., Paulsen N., Tourish D., DiFonzo N. (2004). Uncertainty during organizational change: Is it all about control?. Eur. J. Work. Organ. Psychol..

[38] Hobfoll S.E., Johnson R.J., Ennis N., Jackson A.P. (2003). Resource loss, resource gain, and emotional outcomes among inner city women. J. Pers. Soc. Psychol..

[39] Unsworth K.L., Mason C.M. (2012). Help yourself: The mechanisms through which a self-leadership intervention influences strain. J. Occup. Health Psychol..

[40] Bandura A. (1977). Self-efficacy: Toward a unifying theory of behavioral change. Psychol. Rev..

[41] Wang Y., Huang J., You X. (2016). Personal resources influence job demands, resources, and burnout: A one-year, three-wave longitudinal study. Soc. Behav. Pers..

[42] Reis D., Hoppe A., Schröder A. (2015). Reciprocal relationships between resources, work and study engagement, and mental health: Evidence for gain cycles. Eur. J. Work. Organ. Psychol..

[43] Tims M., Bakker A.B., Derks D. (2012). Development and validation of the job crafting scale. J. Vocat. Behav..

[44] Bakker A.B., Costa P. (2014). Chronic job burnout and daily functioning: A theoretical analysis. Burn. Res..

[45] Vera M., Salanova M., Lorente L. (2012). The predicting role of self-efficacy in the Job Demands-Resources model: A longitudinal study. Estud. Psicol..

[46] Lesener T., Gusy B., Wolter C. (2019). The job demands-resources model: A meta-analytic review of longitudinal studies. Work Stress.

[47] Anseel F., Lievens F., Schollaert E., Choragwicka B. (2010). Response rates in organizational science, 1995–2008: A meta-analytic review and guidelines for survey researchers. J. Bus. Psychol..

[48] Newman D.A. (2014). Missing data: Five practical guidelines. Organ. Res. Methods.

[49] DeSimone J.A., Harms P.D. (2018). Dirty data: The effects of screening respondents who provide low-quality data in survey research. J. Bus. Psychol..

[50] De Lange A.H. (2005). What about Causality? Examining Longitudinal Relations between Work Characteristics and Mental Health.

[51] Enders C.K., Bandalos D.L. (2001). The relative performance of full information maximum likelihood estimation for missing data in structural equation models. Struct. Equ. Model..

[52] James L.A., James L.R. (1989). Integrating work environment perceptions: Explorations into the measurement of meaning. J. Appl. Psychol..

[53] Tomas J., Maslić Seršić D., De Witte H. (2019). Psychological climate predicting job insecurity through occupational self-efficacy. Pers. Rev..

[54] Rigotti T., Schyns B., Mohr G. (2008). A short version of the occupational self-efficacy scale: Structural and construct validity across five countries. J. Career Assess..

[55] Berwick D.M., Murphy J.M., Goldman P.A., Ware J.E., Barsky A.J., Weinstein M.C. (1991). Performance of a five-item mental health screening test. Med. Care.

[56] Tisu L., Lupșa D., Vîrgă D., Rusu A. (2020). Personality characteristics, job performance and mental health: The mediating role of work engagement. Pers. Individ. Differ..

[57] Hellgren J., Sverke M. (2003). Does job insecurity lead to impaired well-being or vice versa? Estimation of cross-lagged effects using latent variable modelling. J. Organ. Behav..

[58] Chen C.-Y., Mao H.-Y., Hsieh A.-T. (2012). Role Ambiguity, employee gender, and workplace friendship. Psychol. Rep..

[59] Han Y., Wang M., Dong L. (2014). Role conflict and the buffering effect of proactive personality among middle managers. Soc. Behav. Pers..

[60] Lu C.-Q., Siu O.-L., Cooper C.L. (2005). Managers’ occupational stress in China: The role of self-efficacy. Pers. Individ. Differ..

[61] Brown T.A. (2015). Confirmatory Factor Analysis for Applied Research.

[62] Muthén L.K., Muthén B.O. (2017). Mplus User’s Guide.

[63] Bentler P.M. (1990). Comparative fit indexes in structural models. Psychol. Bull..

[64] Hu L.-T., Bentler P.M. (1999). Cutoff criteria for fit indexes in covariance structure analysis: Conventional criteria versus new alternatives. Struct. Equ. Model..

[65] Chen F.F. (2007). Sensitivity of goodness of fit indexes to lack of measurement invariance. Struct. Equ. Model..

[66] Lepine J.A., Podsakoff N.P., Lepine M.A. (2005). A meta-analytic test of the challenge stressor-hindrance stressor framework: An explanation for inconsistent relationships among stressors and performance. Acad. Manag. J..

[67] Cavanaugh M.A., Boswell W.R., Roehling M.V., Boudreau J.W. (2000). An empirical examination of self-reported work stress among U.S. managers. J. Appl. Psychol..

[68] Lazarus R.S., Folkman S. (1984). Stress, Appraisal, and Coping.

[69] Ventura M., Salanova M., Llorens S. (2015). Professional self-efficacy as a predictor of burnout and engagement: The role of challenge and hindrance demands. J. Psychol..

[70] Byars-Winston A., Diestelmann J., Savoy J.N., Hoyt W.T. (2017). Unique effects and moderators of effects of sources on self-efficacy: A model-based meta-analysis. J. Couns. Psychol..

[71] Luthans F., Luthans K., Luthans B. (2004). Positive psychological capital: Beyond human and social capital. Bus. Horiz..

[72] Pierce J.L., Gardner D.G. (2004). Self-esteem within the work and organizational context: A review of the organization-based self-esteem literature. J. Manag..

[73] Cartwright S., Cooper C.L. (1997). Managing Workplace Stress.

[74] Sinclair R.R., Allen T., Barber L., Bergman M., Britt T., Butler A., Ford M., Hammer L., Kath L., Probst T. (2020). Occupational Health Science in the Time of COVID-19: Now more than Ever. Occup. Health Sci..

[75] Rigotti T., Yang L.Q., Jiang Z., Newman A., De Cuyper N., Sekiguchi T. (2021). Work-Related Psychosocial Risk Factors and Coping Resources during the COVID-19 Crisis. Appl. Psychol..

