# Supplementary material for: Occupational Self-Efficacy as a Mediator in the Reciprocal Relationship between Job Demands and Mental Health Complaints: A Three-Wave Investigation
Source: Int J Environ Res Public Health. 2021 Nov 2;18(21):11532. doi: 10.3390/ijerph182111532 (PMC8582868; doi:10.3390/ijerph182111532)
Supplement: Supplementary file 1 [file ijerph-18-11532-s001.zip › Supplementary File.pdf]

Note S1. The rationale for the choice of a 6-month time lag.

The choice of a 6-month time lag was based on the available research and on practical considerations unique for this study. Previous studies examining longitudinal relationships between job characteristics and employee well-being utilized time lags ranging from 4 days to 10 years, with a mean of 11 months [46]. Although none of the previous studies suggested the most appropriate time lag needed for demonstrating hypothesized mediation effects (e.g., for the effect from role ambiguity to occupational self-efficacy), the study by de Lange et al. [2] demonstrated that the strongest reciprocal effects between job demands and employee strain can be observed after a period of 1 year (in comparison to 2- and 3-year time lags). Furthermore, time lags shorter than 6 months were deemed a potential risk for seriously reducing employee response rate due to research fatigue (some of the organizations had already been using on-line surveys for internal purposes). Finally, because data collection was conducted in the dynamic private sector, longer time lags than ones used in this study might have increased the probability of unobserved events that could obscure cross-lagged effects or cause dropout of entire organizations (e.g., due to mergers).

Note S2. Description of techniques used to increase response rates.

HR managers provided all employees with advance notice about the study via the organizations' intranet. Where possible, HR managers also announced the survey personally. Second, employees were informed about the university sponsorship of the survey (i.e., it was explained that the survey was part of a collaboration between the organization and the university), a procedure that might give the survey a more neutral and trustworthy image [47]. Third, anonymity was ensured by instructing employees to create unique codes that could not be linked to them personally, but that were necessary to link the surveys across waves. Fourth, reminders were sent to participants in each measurement wave.

Table S1. Sample’s Demographic and Work-related Characteristics.

Variables	n	Percentage
Gender		
Male	457	49.80%
Female	460	50.20%
Education		
Upper secondary or pre-university education	337	36.80%
Bachelor's, Master's or postgraduate degree	580	63.20%
Type of contract		
Permanent	839	91.50%
Temporary	76	8.30%
Occupational training without entering into	2	0.20%
Managerial position		
No	647	70.60%
Yes	270	29.40%
Working hours		
Full-time	914	99.70%
Part-time	3	0.30%

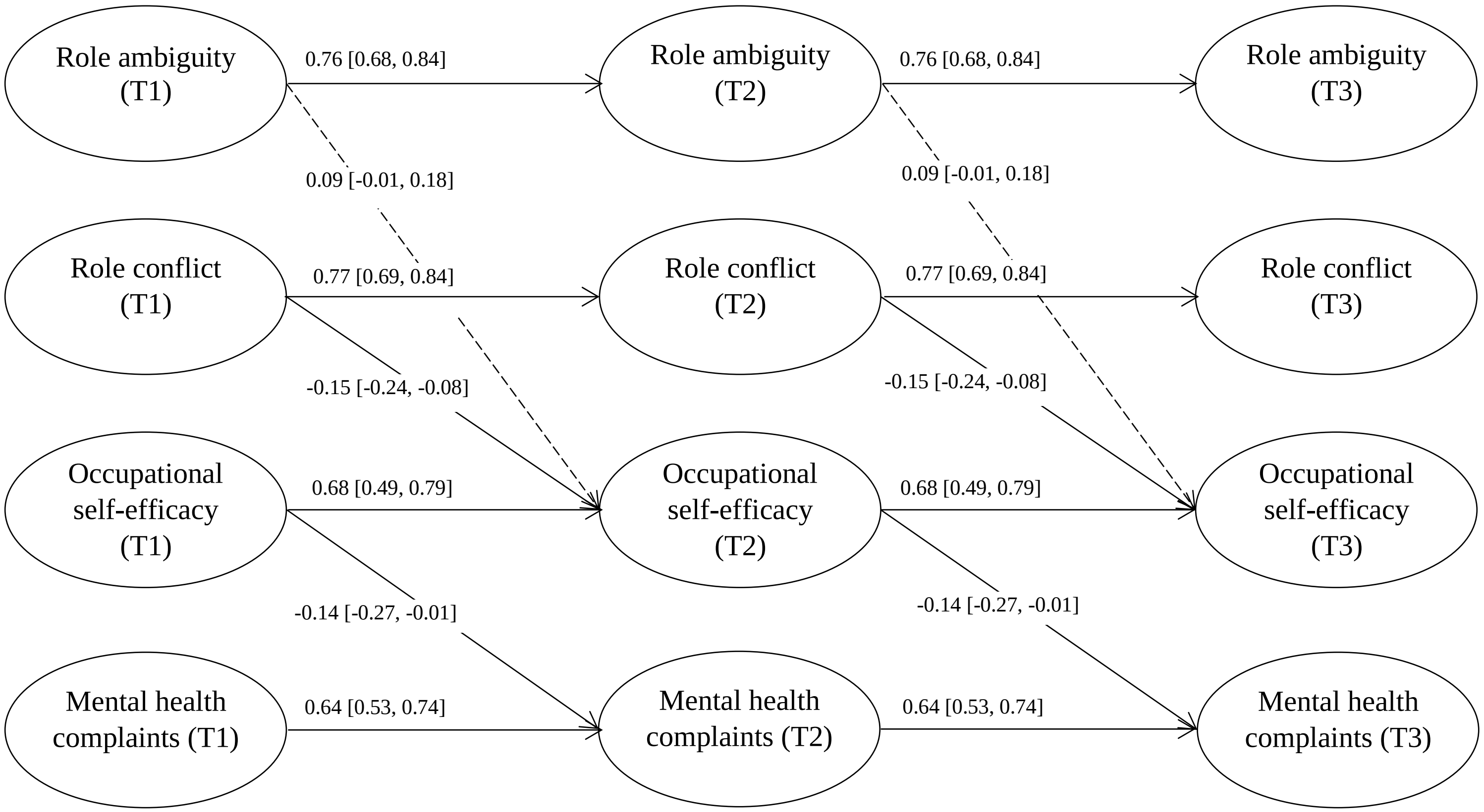

Figure S1. Unstandardized path coefficients and confidence intervals.
The following are not presented for figure clarity: control variables, factor indicators, correlations between latent variables and the direct effects between two role stressors at T1 and mental health complaints T3 (all non-significant). Solid lines present significant effects, dashed lines present non-significant effects.
